# Primary prevention of diabetes mellitus type 2 and cardiovascular diseases using a cognitive behavior program aimed at lifestyle changes in people at risk: Design of a randomized controlled trial

**DOI:** 10.1186/1472-6823-8-6

**Published:** 2008-06-24

**Authors:** Jeroen Lakerveld, Sandra DM Bot, Marijke J Chinapaw, Maurits W van Tulder, Patricia van Oppen, Jacqueline M Dekker, Giel Nijpels

**Affiliations:** 1 EMGO Institute, VU University Medical Center, Amsterdam, The Netherlands; 2 Department of General Practice, VU University Medical Center, v.d. Boechorststraat 7, 1081 BT Amsterdam, The Netherlands; 3 Department of Public and Occupational Health, VU University Medical Center, Amsterdam, The Netherlands; 4 Institute of Health Sciences, Faculty of Earth & Life Sciences, VU University Amsterdam, The Netherlands; 5 Department of Psychiatry, VU University Medical Center, Amsterdam, The Netherlands

## Abstract

**Background:**

The number of people with cardiovascular disease (CVD) and diabetes mellitus type 2 (T2DM) is growing rapidly. To a large extend, this increase is due to lifestyle-dependent risk factors, such as overweight, reduced physical activity, and an unhealthy diet. Changing these risk factors has the potential to postpone or prevent the development of T2DM and CVD. It is hypothesized that a cognitive behavioral program (CBP), focused in particular on motivation and self-management in persons who are at high risk for CVD and/or T2DM, will improve their lifestyle behavior and, as a result, will reduce their risk of developing T2DM and CVD.

**Methods:**

12,000 inhabitants, 30-50 years of age living in several municipalities in the semi-rural region of West-Friesland will receive an invitation from their general practitioner (n = 13) to measure their own waist circumference with a tape measure. People with abdominal obesity (male waist ≥ 102 cm, female waist ≥ 88 cm) will be invited to participate in the second step of the screening which includes blood pressure, a blood sample and anthropometric measurements. T2DM and CVD risk scores will then be calculated according to the ARIC and the SCORE formulae, respectively. People with a score that indicates a high risk of developing T2DM and/or CVD will then be randomly assigned to the intervention group (n = 300) or the control group (n = 300).

Participants in the intervention group will follow a CBP aimed at modifying their dietary behavior, physical activity, and smoking behavior. The counseling methods that will be used are *motivational interviewing *(MI) and *problem solving treatment *(PST), which focus in particular on intrinsic motivation for change and self-management of problems of the participants. The CBP will be provided by trained nurse practitioners in the participant's general practice, and will consists of a maximum of six individual sessions of 30 minutes, followed by 3-monthly booster sessions by phone. Participants in the control group will receive brochures containing health guidelines regarding physical activity and diet, and how to stop smoking. The primary outcome measures will be changes in T2DM and CVD risk scores. Secondary outcome measures will be changes in lifestyle behavior and cost-effectiveness and cost-utility ratios. All relevant direct and indirect costs will be measured, and there will be a follow-up of 24 months.

**Discussion:**

Changing behaviors is difficult, requires time, considerable effort and motivation. Combining the two counseling methods MI and PST, followed by booster sessions may result in sustained behavioral change.

**Trial registration:**

Current Controlled Trials ISRCTN59358434

## Background

Partly because of the ageing population, and partly due to changes in lifestyle and the resulting epidemic of obesity, there is an increasing percentage of people with diabetes mellitus type 2 (T2DM) and cardiovascular diseases (CVDs) in the general population. With a prevalence of 475,000 patients with diabetes and 65,000 new cases each year, diabetes mellitus is one of the most widespread chronic diseases in the Netherlands [1]. CVD is a major cause of disability and mortality in Western countries, and is responsible for one fifth of all disability-adjusted life years [2].

To a large extend, T2DM and CVD are caused by lifestyle dependent risk factors, such as overweight, reduced physical activity, smoking and an unhealthy diet [3-5]. Changing these risk factors has the potential to postpone or prevent the development of T2DM and CVD.

People who are overweight or obese often have a cluster of risk factors for T2DM and CVD, and the risk increases progressively as their body mass index (BMI) increases [6]. Apart from overall obesity, fat distribution is an important risk factor for T2DM and CVD [7]. Waist circumference reflects the magnitude of abdominal adipose tissue deposits as well as total fat mass, and thereby complements BMI in the evaluation of obesity-associated T2DM and CVD risk [8,9]. It is considered to be a good measure with which to identify persons with an increased risk of developing T2DM and CVD [10-12].

The Diabetes Prevention Program Research Group have shown that intensive lifestyle interventions that encourage people to achieve and maintain weight-loss are effective in lowering the incidence of T2DM [13]. In the Finnish Diabetes Prevention Study, a lifestyle intervention in middle-aged, overweight persons resulted in a 58% reduction or postponement of the overall incidence of diabetes [14]. A Dutch RCT showed that a lifestyle-intervention program according to general recommendations (i.e. regular dietary advice, and stimulation to lose weight and to increase physical activity) was effective and induced beneficial changes in lifestyle, which improved glucose tolerance in subjects with impaired glucose tolerance [15].

A wide variation of lifestyle interventions are reported in the literature. Components of interventions that have been found to be effective are: goal-setting [16], programs specifically tailored to the participants [14,17], the use of written materials [14,18], and continued subject-therapist contact, whether in person or by telephone, mail or e-mail [19]. Furthermore, effective interventions included those based on behavioral or cognitive-behavioral strategies, as opposed to health education or instruction alone [18-21].

### Theoretical background

A theory that has been proven useful for the development of behavioral interventions is the theory of planned behavior (TPB) [22,23]. The TPB helps us to understand how energy balance-related behavior is mediated by cognitive constructs [24]. According to the TPB, human action is guided by three kinds of considerations: a) behavioral beliefs: beliefs about the likely consequences of the behavior and the evaluation of these consequences; b) normative beliefs: beliefs about the normative expectations of others and the motivation to comply with these expectations; c) control beliefs: beliefs about the presence of factors that may facilitate, or may impede the performance of the behavior, and the perceived power of these factors. These beliefs can lead, respectively, to a favorable or an unfavorable attitude towards a certain behavior, perceived social pressure, and perceived behavioral control, and in combination they determine behavioral intention. Finally, given a sufficient degree of actual control over the behavior, people are expected to carry out their intentions when the opportunity arises.

Leventhal's theory of self-regulation proposes that individuals construct schematic representations of illness and health-threatening conditions according to the concrete and abstract sources of information that are available to them [25]. The theory starts with the premise that individuals are active problem-solvers. They make sense of a threat to their health by developing their own cognitive representations of the threat, which, in turn, determine how they respond. Health professionals such as nurse practitioners can be sources of information. The theory also assumes that a discrepancy between a person's goal or expected outcome and what exists (the actual situation) will motivate the person to take action and to reduce this ambivalence. Feedback on the discrepancy motivates a person to become actively engaged by attending to stimuli and making efforts to find a way to overcome the discrepancy between what is expected or desired and the present state [26].

### Cognitive behavioral program

The cognitive behavioral program (CBP) applies several components of both the TPB and the theory of self-regulation: The counseling techniques that are used in the intervention are motivational interviewing (MI) and problem-solving treatment (PST). MI aims to reinforce the attitude and the behavioral intention according to the TPB, and induces the ambivalence described in the theory of self-regulation. PST will be used to support the participant in finding ways to overcome this discrepancy. A further aim of the PST is to strengthen the participant's perceived behavioral control and to provide the tools to overcome barriers that hinder a structural change in lifestyle behavior.

MI is a client-centered counseling method with which to elicit behavioral change by helping people to explore and resolve ambivalence in a respectful counseling atmosphere [27]. It is a well-known method of counseling, and is considered to be a useful intervention strategy in the treatment of lifestyle problems [28]. The therapeutic relationship is more like a partnership or companionship than a setting with expert/recipient roles. Motivation is an integral part of changing an individual's behavior and stimulating the adaptation to good healthy habits. The four guiding principles of MI are: express empathy, develop discrepancies, roll with resistance, and support self-efficacy. MI may be a suitable counseling technique with which to establish personal relevance and awareness, so that a positive attitude towards behavioral change is likely to occur.

PST is a brief, structured psychological intervention, and can be defined as the self-directed cognitive-behavioral process by which a person attempts to identify or discover effective or adaptive solutions for specific problems encountered in everyday life [29]. The effects of PST have been proven to be effective in patients with depression [30,31]. PST may increase a person's ability to solve problems in a structured way and also result in increasing the person's self-management. The treatment involves an active collaboration between participant and therapist, with the participant taking an increasingly active role in planning the treatment and implementing activities between the treatment sessions. The therapist helps participants to gain a sense of mastery over their difficulties. Therefore, PST may be a valuable counseling method. The treatment can be provided by nurse practitioners, is relatively brief (i.e. only three to six 30-minute sessions are needed), and is therefore suitable for primary care.

### Objectives

The aim of the study is to investigate the effects of a CBP, compared with providing written information and brochures only. Primary outcomes are the absolute cardiovascular risk and risk of developing T2DM in people who are at high risk for T2DM and/or CVD. Secondary outcomes are changes in dietary behavior, physical activity, and smoking behavior in people who are at high risk for T2DM and/or CVD.

The cost-effectiveness and cost-utility ratios will also be assessed.

## Methods

### Design of the study

**Figure 1:**
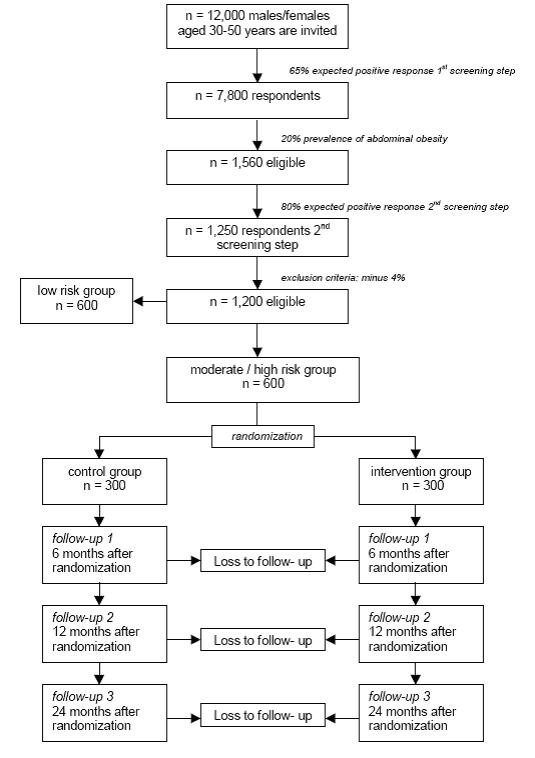
Design of the RCT

The study is designed as a multicenter randomized controlled trial with a two-year follow-up and an economic evaluation alongside. Participants will be recruited through a two-step screening procedure (Figure 1), and those who are eligible and consent will be randomly assigned to the intervention group or the control group. The Medical Ethics Committee of the VU University Medical Center in Amsterdam approved the study design, protocols, information letters and informed consent form.

### Setting

The trial will be conducted in the Diabetes Research Center in Hoorn, in the Netherlands. The intervention will be provided by nurse practitioners in 12 general practices in several municipalities in the semi-rural region (West-Friesland) surrounding the city of Hoorn.

### Study population

Twelve thousand inhabitants (men and women) aged 30-50 living in this region will be invited to participate in a selective screening procedure. The target group will be approached, after identification of date of birth and absence of diabetes or known CVD, from participating general practices (n = 12).

In the first step of the screening procedure the inhabitants will receive an invitation to measure their own waist circumference with a tape measure, according to detailed instructions. Waist circumference is a widely used indicator of abdominal obesity in population studies. To minimize non-response we will use the modified Tailored Design Method [32] to approach potential participants. The main elements of this approach are: a brief pre-notice postcard is sent to each of the sampled persons, and a few days later a comprehensive and easy to understand invitation is sent, together with the tape measure and a stamped addressed envelope for the reply. A week after this mailing, another postcard will be sent, in which the respondents are thanked for returning the survey questions and the non-respondents are asked to complete the waist-measurement and return it immediately. Four weeks later, a reminder and a replacement tape measure are sent to the non-respondents. Abdominal obesity is, in principle, defined as a waist circumference of 88 cm or more for women, and 102 cm or more for men [33]. All respondents with a waist circumference of 1 cm under this cut-off point or above will be invited to participate in the second step of the screening at the research center. After signing an informed consent form, this screening will include the measurement of blood pressure, a blood sample (to determine total and HDL cholesterol, triglycerides, fasting plasma glucose, HbA1c), and anthropometric measurements (height, weight, and waist and hip circumference). In addition, the participants will be given a questionnaire to fill in at home, including questionnaires about physical activity, dietary intake, smoking status, determinants of behavior according to the TPB, quality of life, and depression.

The 9-year risk of developing diabetes will be estimated using the risk function derived from data of the Atherosclerosis Risk In Communities (ARIC) Study [34], with ethnicity (black yes/no), parental history of diabetes, systolic blood pressure, waist circumference and height (Additional file 1).

The 10-year risk of a fatal cardiovascular disease will be estimated using the formulae developed by the Systematic COronary Risk Evaluation (SCORE) project [35], with gender, smoking status, total cholesterol and systolic blood pressure (Additional file 2, see Tables 1 &2).

**Table 1:** Coefficients for Eq. (1)

		CHD		Non-CHD	CVD
				
		α	p	α	p
Low risk	Men	-22.1	4.71	-26.7	5.64
	Women	-29.8	6.36	-31.0	6.62
High risk	Men	-21.0	4.62	-25.7	5.47
	Women	-28.7	6.23	-30.0	6.42

**Table 2:** Coefficients for Eq. (2)

	CHD	Non-CHD
Current smoker	0.71	0.63
Cholesterol (mmol/L)	0.24	0.02
Systolic BP (mmHg)	0.018	0.022

For both scores and for each participant, age will be extrapolated to 60 years to address the problem of a high relative but low absolute risk in younger persons. This way it is possible to flag persons who will become at high absolute risk [36].

All participants with a minimum risk of 10% for developing diabetes and/or a minimum risk of 10% for CVD will be randomly assigned to either the intervention group or the control group. Exclusion criteria are: inability to communicate adequately in the Dutch language and pregnancy.

### Treatment allocation

An allocation schedule will be drawn up with a computerized random number generator. If there is more than one participant from a family, the consecutive members of that family will be allocated to the same group to avoid contamination. Randomization will be performed by an independent administrative assistant from the Diabetes Research Center, who is not involved in the intervention.

### Intervention

#### Intervention group

The participants in the intervention group will receive the CBP to increase their motivation and ability to change their dietary pattern, physical activity or smoking behavior. The CBP will be provided by nurse practitioners in the participating general practices, and will consist of a maximum of 6 individual 30-minute counseling sessions [37], followed by 3-monthly booster sessions by phone for a period of one year. The MI and PST counseling methods will be used. The counseling sessions are initially given on a weekly base, but after a few sessions the time between the sessions is extended to 2/3 weeks, followed by 3-monthly booster sessions by phone, to act as a reminder for the participants, to reinforce what they have learned, and to give them support and feedback.

One of the techniques that is used during MI is reflective listening, which is applied throughout the whole counseling process. A 'gap' between the participant's current behavior and the broader goals will be emphasized, cultivating motivation for lifestyle change. When the participant recognizes such discrepancies, a certain level of discontent arises that makes change more likely to occur. Discrepancies are emphasized after exploring the participant's views on their current and future behaviors. Rolling with resistance invites the participant to consider a new perspective, instead of having it imposed. Finally, self-efficacy, or the confidence to change a specific behavior under difficult circumstances, will be supported whenever possible because it is one of the best predictors of treatment outcome. After the MI counseling the participant is willing to change and it is time to strengthen the commitment to a plan for lifestyle change using PST. The nurse practitioner, together with the participant, will make an implementation plan concerning where, when and how the behavior changes will take place. This will be done in the following series of stages [38]: explanation of the intervention and its rationale, definition and breaking down of the problem, establishing achievable goals for problem-solving (achievable goals are SMART goals: Specific, Measurable, Achievable, Relevant, Timed), generating multiple possible solutions, evaluating and choosing the solution, implementing the preferred solution, and evaluating the outcome.

Each participant will be free to choose which lifestyle component(s) (smoking, physical activity or diet) he or she wants to change. The CBP focuses on one problem area, for which multiple sessions might be needed. MI will be used first, followed by PST when the participant is motivated. During the intervention it might be necessary to revert to MI to motivate the participant again, after which new goals can be set. The last CBP session is brought to an end by reviewing and emphasizing the entire CBP process, with emphasis on the successes achieved by the participant and implementation of CBP in the future, independent of the nurse practitioner.

#### Control group

The participants in the control group will receive written information about their risk of developing T2DM and CVD, and existing brochures containing health guidelines regarding physical activity, a healthy diet, and how to stop smoking.

Participants with a systolic blood pressure above 160 mmHg and/or hypercholesterolemia (> 8 mmol/L) will be referred to their GP for additional medication, but will remain in the study. Participants with a glucose value above 7.0 mmol/L (confirmed with a second blood sample) will also be referred to their GP and then excluded for participation.

#### Training of nurse practitioners

Prior to the onset of the study, the nurse practitioners will receive a total of 18 hours of training from experienced psychologists R. Bes (member of the Motivational Interviewing Network of Trainers (MINT)) and J. Tijhaar (PST trainer) who are specialized in providing and co-ordinating CBPs. A treatment manual will be used during the study to guide the treatment. During the intervention the nurse practitioners will receive ongoing support during regular peer supervision meetings. Random CBP sessions will be tape-recorded to assess the adherence of the nurse practitioners to the protocol. These tape recordings will also be used in the peer supervision meetings to provide ongoing feedback and to increase uniformity of the counseling style among the nurse practitioners.

### Blinding

It is impossible to blind the participants and nurse practitioners for the intervention, but the research assistants, the principal investigator and the GPs will remain blinded during the entire intervention. This is achieved by instructing the participants not to communicate about the intervention with their GP or the medical assistants.

### Study duration

The counseling sessions will last for 2-4 months, and the total intervention period, including booster calls, will be 16 months. Together with an estimated inclusion period of 8 months, the study itself will take 24 months (2 years). The measurements will be carried out before the start of the intervention, and after 6, 12 and 24 months. The baseline measurements started on February 14^th^, 2008.

### Primary outcome measurements

- Diabetes risk score and cardiovascular risk score.

The risk of developing diabetes will be assessed by means of a risk function, calculated according to data from the ARIC Study [34] (see Additional file 1). Absolute cardiovascular risk will be assessed with a risk score, calculated according to the SCORE project [35] (see Additional file 2). For both sores and for each participant, age is set at 60 years.

### Secondary outcome measurements

- Dietary behavior, physical activity behavior, and smoking behavior.

- Cost-effectiveness and cost-utility of the CBP.

All relevant direct, indirect and total costs will be included. Costs related to diabetes, diabetic complications and cardiovascular disease are considered to be relevant. The costs of the CBP will be assessed using the bottom-up procedure, based on personnel, material and housing costs.

### Data-collection

#### Questionnaires

▪ Dietary behavior: An extended and modified version of the 8-item Food Frequency Questionnaire [39] will be used to measure the intake of fruit, vegetable, fiber, alcohol consumption and (un)saturated fat. Total energy intake can not be measured with this questionnaire.

▪ Physical activity behavior: The Short Questionnaire to Assess Health Enhancing Physical Activity (SQUASH) [40] will be used to assess physical activity within (A) commuting activities, (B) leisure time activities, (C) household activities, and (D) activities at work and school.

▪ Sedentary behavior during leisure time will be measured with a subscale of the Activity Questionnaire for Adolescents & Adults (AQUA) [41].

▪ Smoking behavior: Smoking every day/smoking now and then/never smoked/year of smoking cessation and pack years (average number packs of 20 cigarettes per day smoked, multiplied by the number of years as a smoker).

▪ Determinants of behavioral change: A 41-item questionnaire, based on existing TPB questionnaires, will be used to assess lifestyle-specific behavior with regard to the constructs attitude, social norm, self-efficacy and intention to change. To our knowledge, no suitable validated questionnaires are available. The theoretical model and questionnaires in other current studies at the research institute were used to develop this Determinants of Lifestyle Behavior Questionnaire (DLBQ).

▪ Quality of life will be assessed with the EuroQol (EQ-5D) [42,42,43].

▪ Compliance: The number of CBP sessions attended will be registered, and compliance is considered adequate if at least two sessions are attended.

#### Cost-diaries

▪ Direct health care, non-health care costs and indirect non-health care costs will be recorded in cost-diaries that will be sent via e-mail to the participants in 3-monthly time-intervals. Printed cost-diaries will be sent to participants who do not have access to or dislike the Internet. Direct health care costs will include costs of the CBP, visits to the GP, medical specialists, therapists, dieticians, medication, and hospitalization. Direct non-health care costs will include the costs of over-the-counter medication, physical activity programs, travel time, and waiting time. Indirect costs will include the costs of absence from paid and unpaid work.

▪ Co-interventions: In both groups, any co-intervention will be reported in the cost-diaries.

#### Physical measurements

▪ Anthropometric measurements: Weight, height, and waist-hip circumferences. Body weight and height will be measured to calculate the BMI (weight divided by height squared). Height will be measured to the nearest 0.1 cm without shoes, whereas weight will be measured to the nearest 0.5 kg, wearing indoor clothing and no shoes. Waist circumference will be measured at the level midway between the lowest rib margin and the iliac crest, and the hip circumference at the widest level over the greater trochanters.

▪ Systolic and diastolic blood pressure will be measured after 5 minutes of rest, in a seated position, with the Collin Press Mate (BP-8800, Komaki-City).

▪ Laboratory tests: Fasting blood samples will be taken (venapunction) to measure glucose, Hb1Ac, total cholesterol, HDL-cholesterol, and triglycerides.

### Process evaluation

A process evaluation will be carried out to study the effective and ineffective parts of the intervention. In the second questionnaire the participants of the intervention group are asked to score three items regarding their satisfaction with the intervention on a 4-point Likert scale, and indicate whether or not they are changing their lifestyle as planned in the program (yes/no). The nurse practitioners are asked to give their opinion on a 4-point Likert scale regarding five statements (i.e. about the MI and PST training and their confidence in providing the counseling sessions), and on two open-ended questions about their perception of the effectiveness of the training. These statements are scored twice: directly after the training session and again after the recruitment period (about eight months later). The quality of the nurse practitioner MI will be assessed according to the Motivational Interviewing Treatment Integrity (MITI), a behavioural coding system that will be applied to the tape-recordings of the counseling sessions [44]. The results will be linked with the risk scores of the participants after one year. This will make it possible to evaluate the effect of the counseling skills of the nurse practitioners on patient outcomes.

### Sample size

In a Dutch working population of overweight people (BMI ≥ 25), which consisted of both males and females, the standard deviation of the ARIC score was found to be 8.11 [45]. For a two-sided detection of a 5% between-group difference in cardiovascular risk score, standardized at 60 years of age, with an alpha of 0.05 and a power of 90%, in the present study 120 participants will be needed in the intervention group and 120 in the control group. Assuming that 40 participants per collaborating GP are recruited, and each participant is randomized independently, this is feasible. We expect a response of approximately 65% in the first step of the screening, 20% of which will meet the abdominal obesity criterion [46,47]. Of those who meet this criterion, we expect that at least 80% will be willing to participate in the second step and the RCT (Figure 1).

A multilevel analysis is performed to be able to adjust for the clustering of observations within practices. To be able to perform stratified and multivariable analyses, and taking loss to follow-up into account, larger numbers will be needed. A total of 600 (i.e. 300 in the intervention group and 300 in the control group) is expected to suffice.

### Analyses

Descriptive statistics (means ± SD, or median and interquartile ranges, as appropriate) will be used to describe the entire study sample with regard to demographics, physical measurements and baseline lab values. The analyses will be conducted according to the intention to treat principle. Multilevel analyses will be performed to adjust for the clustering of observations of participants receiving care from the same nurse practitioner, and for repeated measurements within one patient. Linear and logistic regression models will be used to examine the effect of the intervention on each of the outcome measurements. Differences in changes between groups will be calculated with 95% confidence intervals. If there are any relevant differences in baseline measurements between the two groups, we will adjust the outcome for these factors (i.e. age, gender, ethnicity, BMI at the start of study, level of education, depression, smoking, number of sessions attended). Separate analyses of effect modifiers (i.e. gender, age) will be performed in order to gain a better understanding as to who benefits most from the intervention. Separate analyses of effect modifiers and mediators will be conducted in order to gain a better understanding as to who benefits most from the intervention

### Economical evaluation

An economic evaluation will be performed after 24 months from the societal perspective and from the health insurance perspective. Differences in mean costs between the two groups will be presented with 95% confidence intervals, estimated with bootstrapping methods. Incremental cost-effectiveness ratios (ICERs) will be calculated by dividing the incremental mean costs by the incremental mean effects for: a) reduction in the risk of developing diabetes based on the ARIC score, b) reduction in the risk of future cardiovascular disease based on the SCORE, and c) quality-adjusted life years (QALYs) based on the EuroQol. The 'Dutch EQ-5D tariff' [48] will be used to calculate utilities.

Bootstrapping methods will be used to derive cost-effectiveness planes and acceptability curves.

## Discussion

This article presents a detailed description of an RCT, designed with the aim to investigate the effectiveness of a CBP to improve lifestyle behavior and reduce T2DM and CVD risk in a high-risk population. This will provide researchers, health care providers and policy makers the opportunity to critically review the methodological quality, the background theory and the practical issues of the RCT. An innovative and key element of this trial is that the participants will be self-empowered to accomplish a long-lasting change in their lifestyle. We expect that the specific counseling methods (MI and PST) will induce an effective, sustained reduction in T2DM and CVD risk. Both counseling methods are practical, evidence-based tools. The combination of MI and PST, in particular, is used before [49], but never in preventive trials. Combining the two counseling methods may be effective because PST can help to resolve the ambivalence created with MI. By doing so, practical tools to sustained pro-active behavioral change are handed to the participants, who become aware of their unhealthy behavior and are intrinsically motivated to change. The participants will be free to choose which lifestyle component(s) (smoking, physical activity or diet) they want to change. Offering different behavioral alternatives to achieve the participant's goals may positively affect compliance and long-term success.

After the counseling the participants will receive continuing support in the form of 3-monthly booster sessions by phone, which will help them to sustain behavioral changes.

There are some limitations in the study design. Critics may raise concerns about the use of the DLBQ to assess determinants of change in lifestyle behavior, because this questionnaire has not been validated. We developed this questionnaire by using several questionnaires of colleagues in the field since we were not able to find a validated questionnaire to assess determinants of behavior. Another concern could be the benefit participants in the intervention group may have from the extra attention they get. However, attention alone is unlikely to result in behavioral change. Furthermore, it has been demonstrated that MI and PST are significantly more effective than attention alone [50-52].

Non-respondents are a potential threat to the external validity of a study. Therefore we chose to approach potential participants with correspondence at multiple moments, as described by Dillman et. al. [32]. The intervention will take place in close proximity to the homes of the participant, and this could reduce barriers for participation. Furthermore, two bicycles will be raffled among those who respond to the first screening step. Despite of these measures, we expect that participants who agree to participate are higher educated and more willing to change their lifestyle than the non-respondents [53]. However, because the participants will be randomized, this will be the case in both groups. Having a population of motivated participants might decrease the drop-out percentage.

A strength of the study design is its focus on lifestyle dependent risk factors that are associated with both T2DM and CVD. To select people who are at risk of these diseases, self-measured waist circumference is used as a first screening step, which is a simple and practical method. Another strength is the careful monitoring of the intervention by providing standardized training for the nurse practitioners and feedback on the counseling sessions by means of tape recordings.

If this intervention has strong positive effects, the CBP could be widely implemented. The process evaluation of our intervention will come up with barriers and facilitators that can be used for the implementation strategy.

## Abbreviations

ARIC: atherosclerosis risk in communities; AQUA: activity questionnaire for adolescents & adults; BMI: body mass index; CBP: cognitive behavioral program; CVD: cardiovascular diseases; DLBQ: determinants of lifestyle behavior questionnaire; GP: general practitioner; ICERs: incremental cost-effectiveness ratios; MI: motivational interviewing; MITI: motivational interviewing treatment integrity; PST: problem solving treatment; RCT: randomized controlled trial; T2DM: diabetes mellitus type 2; TPB: theory of planned behavior; QUALYs: quality adjusted life years; SQUASH: short questionnaire to assess health enhancing physical activity.

## Competing interests

The authors declare that they have no competing interests.

## Authors' contributions

JL is responsible for the data-collection and wrote, together with SDMB, the manuscript. SDMB, PvO, JMD, MJC and GN developed the original concept for the study. The study design was further developed by JL, SDMB, MJC, MWvT, and GN. All authors have read and approved the final manuscript.

## Pre-publication history

The pre-publication history for this paper can be accessed here:



## Supplementary Material

Additional file 1 **Calculating 9-year risk estimates of developing diabetes **[34]The following are parameter estimates for the models estimating the probability of developing diabetes over a 9-year follow-up period: Pr(DM) = 1/(1 + *e*^-x^), where *x *= *Clinical variables only model*: -7.3359 + 0.0271 × 60 (fixed age) + 0.2295 × black + 0.5463 × parental history of diabetes + 0.0161 × systolic blood pressure (mmHg) + 0.0412 × waist (cm) - 0.0115 × height (cm). Black = 1 if Negroid, 0 if white, and parental history of diabetes = 1 if at least one parent has diabetes or 0 if not. Click here for file

Additional file 2 **Calculating 10-year risk estimates for fatal CVD **[35]. **Step 1**. Calculate the underlying risks for coronary heart disease and for non-coronary cardiovascular disease separately for the person's age now (for this study, age now is set as 60) and for their age in ten years time, using the values for alpha and p shown in Table 1. The underlying survival probability, S0, is given by: *S*_*0*_*(60) *= exp{-(exp(α))(60 - 20)^p^}. *S*_*0*_(*70*) = exp{-(exp(α))(*60 *- 10)^p^}. **Step 2**. Using the coefficients in Table 2, calculate the weighted sum, *w*, of the risk factors cholesterol, smoking and systolic blood pressure. Two weighted sums will have to be calculated, one for coronary heart disease and one for non-coronary cardiovascular disease. Smoking is coded as 1 for current and 0 for non-smoker, so no value for smoking has to be entered if the person is a non-smoker. Cholesterol is measured in mmol/L and SBP is measured in mmHg. The weighting for each risk factor is denoted by beta. *w = β*_*chol*_*(cholesterol *- 6) + *β*_*SBP*_(*SBP *- 120) + *β*_*smoker*_*(current)*. **Step 3**. Combine the underlying risks for coronary heart disease and for non-coronary cardiovascular disease, at the person's age and at their age ten years from now (four calculations) which were calculated in step 1 with the weighted sum of a person's risk factors from step 2 for the two end-points, coronary heart disease and non-coronary cardiovascular disease to get the probability of survival at each age for each cause. *S(60) *= *{S*_*0*_*(60}*^exp(*w*)^. *S(70*) = {*S*_0_*(70*)}^exp(*w*)^. **Step 4**. For each cause, calculate the 10-year survival probability based on the survival probability for the person's current age and their age in 10 years time: *S*_10_(60) = *S(70*)/*S(60)*. **Step 5**. Calculate the 10 year risk for each end-point as *Risk*_10 _= 1 - *S*_10_(*60*). **Step 6**. Combine the risks for coronary heart disease and non-coronary cardiovascular disease by adding them: *CVDRisk*10(60) = *[CHDRisk(60)] *+ [*Non*-*CHDRisk(60)]* Click here for file
