# Supplementary material for: Primary prevention of diabetes mellitus type 2 and cardiovascular diseases using a cognitive behavior program aimed at lifestyle changes in people at risk: Design of a randomized controlled trial
Source: BMC Endocr Disord. 2008 Jun 24;8:6. doi: 10.1186/1472-6823-8-6 (PMC2446389; doi:10.1186/1472-6823-8-6)
Supplement: Additional file 1 — Calculating 9-year risk estimates of developing diabetes [34]The following are parameter estimates for the models estimating the probability of developing diabetes over a 9-year follow-up period: Pr(DM) = 1/(1 + e-x), where x = Clinical variables only model: -7.3359 + 0.0271 × 60 (fixed age) + 0.2295 × black + 0.5463 × parental history of diabetes + 0.0161 × systolic blood pressure (mmHg) + 0.0412 × waist (cm) - 0.0115 × height (cm). Black = 1 if Negroid, 0 if white, and parental history of diabetes = 1 if at least one parent has diabetes or 0 if not. [file 1472-6823-8-6-S1.doc]

## Additional file 1 – Calculating 9-year risk estimates of developing diabetes [34]

The following are parameter estimates for the models estimating the probability of developing diabetes over a 9-year follow-up period:

Pr(DM) = 1/(1 + *e*^–x^), where *x* =

*Clinical variables only model*: –7.3359 + 0.0271 x 60 (fixed age) + 0.2295 x black + 0.5463 x parental history of diabetes + 0.0161 x systolic blood pressure (mmHg) + 0.0412 x waist (cm) – 0.0115 x height (cm).

Black = 1 if Negroid, 0 if white, and parental history of diabetes = 1 if at least one parent has diabetes or 0 if not.
