# Supplementary material for: Primary prevention of diabetes mellitus type 2 and cardiovascular diseases using a cognitive behavior program aimed at lifestyle changes in people at risk: Design of a randomized controlled trial
Source: BMC Endocr Disord. 2008 Jun 24;8:6. doi: 10.1186/1472-6823-8-6 (PMC2446389; doi:10.1186/1472-6823-8-6)
Supplement: Additional file 2 — Calculating 10-year risk estimates for fatal CVD [35]. Step 1. Calculate the underlying risks for coronary heart disease and for non-coronary cardiovascular disease separately for the person's age now (for this study, age now is set as 60) and for their age in ten years time, using the values for alpha and p shown in Table 1. The underlying survival probability, S0, is given by: S0(60) = exp{-(exp(α))(60 - 20)p}. S0(70) = exp{-(exp(α))(60 - 10)p}. Step 2. Using the coefficients in Table 2, calculate the weighted sum, w, of the risk factors cholesterol, smoking and systolic blood pressure. Two weighted sums will have to be calculated, one for coronary heart disease and one for non-coronary cardiovascular disease. Smoking is coded as 1 for current and 0 for non-smoker, so no value for smoking has to be entered if the person is a non-smoker. Cholesterol is measured in mmol/L and SBP is measured in mmHg. The weighting for each risk factor is denoted by beta. w = βchol(cholesterol - 6) + βSBP(SBP - 120) + βsmoker(current). Step 3. Combine the underlying risks for coronary heart disease and for non-coronary cardiovascular disease, at the person's age and at their age ten years from now (four calculations) which were calculated in step 1 with the weighted sum of a person's risk factors from step 2 for the two end-points, coronary heart disease and non-coronary cardiovascular disease to get the probability of survival at each age for each cause. S(60) = {S0(60}exp(w). S(70) = {S0(70)}exp(w). Step 4. For each cause, calculate the 10-year survival probability based on the survival probability for the person's current age and their age in 10 years time: S10(60) = S(70)/S(60). Step 5. Calculate the 10 year risk for each end-point as Risk10 = 1 - S10(60). Step 6. Combine the risks for coronary heart disease and non-coronary cardiovascular disease by adding them: CVDRisk10(60) = [CHDRisk(60)] + [Non-CHDRisk(60)] [file 1472-6823-8-6-S2.doc]

## Additional file 2 – Calculating 10-year risk estimates for fatal CVD [35]

**Step 1**

Calculate the underlying risks for coronary heart disease and for non-coronary cardiovascular disease separately for the person's age now (for this study, age now is set as 60) and for their age in ten years time, using the values for alpha and p shown in Table 1. The underlying survival probability, S0, is given by:

*S_0_(60) =* exp{–(exp(α))(60 *–* 20)^p^}

*S_0_(70*) = exp{–(exp(α))(*60 –* 10)^p^}

**Step 2**

Using the coefficients in Table 2, calculate the weighted sum, *w*, of the risk factors cholesterol, smoking and systolic blood pressure. Two weighted sums will have to be calculated, one for coronary heart disease and one for non-coronary cardiovascular disease. Smoking is coded as 1 for current and 0 for non-smoker, so no value for smoking has to be entered if the person is a non-smoker. Cholesterol is measured in mmol/L and SBP is measured in mmHg. The weighting for each risk factor is denoted by beta.

*w =β_chol_(cholesterol –* 6) + *β_SBP_*(*SBP –* 120) + *β_smo_*_ker_*(current)*

Step 3

Combine the underlying risks for coronary heart disease and for non-coronary cardiovascular disease, at the person's age and at their age ten years from now (four calculations) which were calculated in step 1 with the weighted sum of a person's risk factors from step 2 for the two end-points, coronary heart disease and non-coronary cardiovascular disease to get the probability of survival at each age for each cause.

*S(60) = {S_0_(60}*^exp(^*^w^*)

*S(70*) = {*S*_0_*(70*)}^exp(^*^w^*)

Step 4

For each cause, calculate the 10-year survival probability based on the survival probability for the person's current age and their age in 10 years time:

*S*_10_(60*)* = *S(70*) /*S(60)*

Step 5

Calculate the 10 year risk for each end-point as

*Risk*_10_ = 1 - *S*_10_(*60)*

Step 6

Combine the risks for coronary heart disease and non-coronary cardiovascular disease by adding them:

*CVDRisk*10(60*) = [CHDRisk(60)]* + [*Non*-*CHDRisk(60)]*
